# Validation and comparison of EuroQoL-5 dimension (EQ-5D) and Short Form-6 dimension (SF-6D) among stable angina patients

**DOI:** 10.1186/s12955-014-0156-6

**Published:** 2014-10-25

**Authors:** Jing Wu, Yuerong Han, Fei-Li Zhao, Jin Zhou, Zhijun Chen, He Sun

**Affiliations:** School of Pharmaceutical Science and Technology, Tianjin University, No.92 Weijin Rd, Nankai District Tianjin, 300072 P R China; Clinical Pharmacology and Toxicology, University of Newcastle, University Drive, Callaghan, NSW 2308 Australia; Tianjin Chest Hospital, No 93, Xi’an Road, Heping District Tianjin, China; Affiliated Hospital of Logistics University of Chinese People’s Armed Police Force, 220 Chenglin Rd, Dongli District Tianjin, China

**Keywords:** Quality of life, Stable angina, EQ-5D, SF-6D, Utility, China

## Abstract

**Objectives:**

Several preference-based health-related quality of life (HRQoL) instruments have been published and widely used in different populations. However no consensus has emerged regarding the most appropriate instrument in therapeutic area of stable angina. This study compared and validated the psychometric properties of two generic preference-based instruments, the EQ-5D and SF-6D, among Chinese stable angina patients.

**Methods:**

Convergent validity of the EQ-5D and SF-6D was examined with eight a priori hypotheses from stable angina patients in conjunction with Seattle Angina Questionnaire (SAQ). Responsiveness was compared using the effect size (ES), relative efficiency (RE) and receiver operating characteristic (ROC) curves. Agreement between the EQ-5D and SF-6D was tested using intra-class correlation coefficient (ICC) and Bland-Altman plot. Factors affecting utility difference were explored with multiple linear regression analysis.

**Results:**

In 411 patients (mean age 68.08 ± 11.35), mean utility scores (SD) were 0.78 (0.15) for the EQ-5D and 0.68 (0.12) for the SF-6D. Validity was demonstrated by the moderate to strong correlation coefficients (Range: 0.368-0.594, P< 0.001) for five of the eight hypotheses in both the EQ-5D and SF-6D. There were no serious floor effects for the EQ-5D and SF-6D, but ceiling effects for the EQ-5D were large. The areas under ROC of them all exceeded 0.5 (0.660-0.814, P< 0.001). The SF-6D showed a better discriminative capacity (ES: 0.573 to 1.179) between groups with different stable-angina-specific health status than the EQ-5D (ES: 0.426 to 1.126). RE suggested that the SF-6D (RE: 44.8 to 177.8%) was more efficient than the EQ-5D except for physical function. Poor agreement between them was observed with ICC (0.448, P< 0.001) and Bland-Altman plot analysis. Multiple liner regression showed that clinical variables significantly (P< 0.05) influenced differences in utility scores between the EQ-5D and SF-6D.

**Conclusions:**

Both EQ-5D and SF-6D are valid and sensitive preference-based HRQoL instruments in Chinese stable angina patients. The SF-6D may be a more effective tool with lower ceiling effect and greater sensitivity. Further study is needed to compare other properties, such as reliability and longitudinal response.

## Background

There is an increasing demand for cost-utility analysis (CUA), which allows decision-makers to compare the value of interventions for different health problems and has been adopted by many countries such as the UK and US [1,2]. The most commonly used outcome indicator in CUA is the quality adjusted life year (QALY) which is a combination of the time spent in a health state and a utility value representing quality of life for that particular health state [3]. Utility values usually range from 1 (full health) to 0 (death) and direct methods of measuring utilities (e.g. standard gamble or time trade-off) are complex and time-consuming. As an alternative, preference-based instruments are increasingly used in clinical studies and population surveys to generate utility scores [4]. They allow each health status to be described using a simple health status classification system, which can then be used to calculate utility scores with a validated algorithm [5]. Several preference-based instruments including the Quality of Well Being (QWB) [6], Health Utilities Index (HUI) [7], EQ-5D [8], Assessment of Quality of Life (AQoL) [9] and the SF-6D [10] have been published and widely used in different populations.

Given its low respondent burden, the EQ-5D has gained widespread use in clinical studies and population surveys. The EQ-5D has a number of country-specific choice-based preference weights, including weights for the UK, the US, Canada [11], Japan [7] and China [12]. In the UK, National Institute for Health and Clinical Excellence (NICE) currently suggests that the most preferred preference-based instrument is the EQ-5D but recognizes that the EQ-5D may not be appropriate in all circumstances [1]. The SF-6D, which is derived from the 36-item Short Form Health Survey (SF-36), is one of the most widely used generic measures of HRQoL in clinical trials. The major reason for developing SF-6D is to considerably extending the scope for undertaking economic evaluation in health care using existing and future SF-36 data sets [10]. Several studies have compared EQ-5D with SF-6D in different patient groups, including chronic prostatitis [13], chronic heart failure [14], coronary heart disease [15], chronic pain [16], type 2 diabetes [17], inflammatory arthritis [18] and mental health [19]. Fei-Li Zhao et al. found that both EQ-5D and SF-6D are demonstrated to be valid and sensitive HRQoL measures in Chinese chronic prostatitis patients, with SF-6D showing better HRQoL dimension coverage, greater sensitivity, and lower ceiling effect [13]. While, Marko Obradovic et al. found that EQ-5D scores were lower than SF-6D scores in patients with chronic pain, with EQ-5D showing higher construct validity and responsiveness [16]. In general, the two measures are not equivalent and the validity and comparative responsiveness of the EQ-5D and SF-6D differ depending on the population [13–19]. The choice of instrument to measure HRQoL may have potential implications for decision-making [18]. Evidence comparing the performance of these instruments is needed to inform the selection of the most appropriate instrument. In addition, the evidence requires cumulative results from different settings and types of study [20].

Stable angina, the cardinal symptom of coronary artery disease (CAD), is a major debilitating health condition with common chronic symptoms of intermittent, reversible chest pain or discomfort [21]. In China, approximately 7.7 thousand per million people have CAD and about half of them suffer from angina [22,23]. Stable angina has a major negative impact on health-related quality of life (HRQoL), including poor general health status, pain, impaired role functioning, activity restriction, inability to self-manage, and psychological distress [24]. HRQoL measurement among patients with stable angina is thus important for evaluation of new health technologies and resource allocation decisions. Cardiac trials commonly include the collection of different disease-specific and generic measures of health status, such as the Seattle Angina Questionnaire (SAQ) [25], Angina Pectoris Quality of Life Questionnaire (APQLQ) [26], SF-36 [27], and the Nottingham Health Profile (NHP) [28]. However, these instruments can’t be used to elicit utility values for calculating QALYs, which is a fundamental component in CUA as mentioned above. As the management of stable angina patients could potentially involve substantial resource consumption [29], providing preference-based measures that can be incorporated into economic evaluation is particularly important. Establishing practicality and validity of these measures is required before their application [30]. To the best of our knowledge, no preference-based instrument has been validated among stable angina patients. Therefore, the objective of this study was to evaluate the validity and sensitivity of the EQ-5D and SF-6D on stable angina patients and further to evaluate and compare the performance of these two instruments.

## Methods

### Study design and patient recruitment

A survey was conducted in two cities of China, Tianjin (northern China) and Chengdu (southern China), from July to December, 2011. Stable angina patients were recruited in two tertiary hospitals in Tianjin and two community health service centers (CHS) in Chengdu as chronic illness is managed in communities in Chengdu, but not in Tianjin.

Patients were included in the study if they were 18 years or above and had been clinically diagnosed with stable angina by their attending physicians based on clinical symptoms, examinations of coronary angiography, dual source Computer Tomography (CT), and history of CAD. Additional criterion included typical angina symptoms with a report of at least one episode of chest pain in the previous 3 months. Patients were excluded from participating if they had experienced acute myocardial infarction or coronary revascularization such as coronary artery bypass grafting surgery and percutaneous intervention in the previous 6 months. Patients were also excluded if they had any active exacerbation of gastrointestinal (GI) problems, such as an ulcer, or if they were unable to differentiate between their GI symptoms and angina pain. These criteria were used to help increase the likelihood that patients’ chest pain was cardiac in nature rather than non-cardiac.

The study protocol was approved by the Institutional Review Board (IRB) of Tianjin University, and written informed consent concerning the conduct of the survey was obtained from each subject before participating in the study. Patients were interviewed by a trained interviewer with a standardized questionnaire. The questionnaire contained a set of socio-demographic, disease duration, comorbid conditions (hypertension, diabetes mellitus, and hyperlipidemia), and life style questions followed by the instruments of the SAQ, EQ-5D, EQ-VAS, and SF-6D. The patient-reported outcomes including EQ-5D and SF-6D measures were completed by the patients themselves. The procedure and questionnaire used were identical between the two cities.

### Instruments

The EQ-5D is a brief, multi-attribute, generic, preference-based HRQoL instrument. Its descriptive system covers five dimensions including mobility, self-care, usual activities, pain/discomfort, and anxiety/depression. Each dimension has three response levels (no problem, some problems, and severe problems). The EQ-5D descriptive system generates 243 health states, each of which was assigned a utility score ranging from −0.59 to 1.00 (full health). The utility scoring algorithm adopted in this study was developed using time trade-off (TTO) based preference scores from a China general population [12]. The EQ-5D also includes a 20-cm vertical VAS, with 0 and 100 representing worst and best imaginable health states, respectively. The simplified Chinese version of the EQ-5D/VAS was verified in Chinese population [31,32].

The SF-6D is derived from the SF-36 and covers six dimensions including physical functioning, role limitation, social functioning, pain, mental functioning, and vitality. Each dimension has four to six response levels. Totally the SF-6D system defines 18,000 health states with a utility score ranging from 0.29 to 1.00 [10]. The SF-6D utility scoring algorithm used in this study was derived from a representative sample of the UK general population using the Standard Gambling (SG) method, since no Chinese preferences were available [10]. The Chinese version of the SF-6D was translated by Lam et al. in Hong Kong, which was proven to be feasible, acceptable, reliable, and valid in a Chinese population [33].

The SAQ is a disease-specific instrument for patients with angina with 19-item self-administered questions on five dimensions including exertional capacity scale (ECS), anginal stability scale (ASS), anginal frequency scale (AFS), treatment satisfaction scale (TSS), and the disease perception scale (DPS) [25]. The SAQ is scored by assigning each response an ordinal value, beginning with 1 for the response that implies the lowest level of functioning to 5 that implies the highest level of functioning, and summing across items for each of the 5 dimensions. Scale scores for each dimension are then transformed to a 0 to 100 range by subtracting the lowest possible, dividing by the range of the scales, and multiplying by 100. As each scale monitors a unique dimension, no summary score is generated. The Chinese SAQ has been shown to be a valid, responsive and reliable instrument [34].

### Data analyses

#### Descriptive statistics

Descriptive statistics were performed to characterize the sample and the scores of the EQ-5D/VAS, SF-6D, and SAQ. Continuous variables are presented as mean, standard deviation (SD) and categorical variables are shown in the number and proportion of the sample within each group.

#### Construct validation

Convergent validity of the EQ-5D and SF-6D was assessed by examining their association with the SAQ and EQ-VAS at the domain and scale level. Based on the literature and clinical experience, eight *a priori* hypotheses were generated where moderate-to-strong correlations were expected, namely: 1) the EQ-5D and SF-6D utility scores with SAQ physical limitation; 2) the EQ-5D and SF-6D utility scores with SAQ angina stability; 3) the EQ-5D and SF-6D utility scores with SAQ angina frequency; 4) the EQ-5D and SF-6D utility scores with SAQ treatment satisfaction; 5) the EQ-5D and SF-6D utility scores with SAQ disease perception; 6) the EQ-5D and SF-6D utility scores with the EQ-VAS; 7) the EQ-5D pain/discomfort and SF-6D pain with SAQ angina frequency; 8) the EQ-5D performing usual activities and the SF-6D physical function with the SAQ physical limitation. The correlation was estimated with Spearman’s rank correlation coefficient, with p > 0.5 considered strong correlation, 0.35 to 0.5 considered moderate correlation, and 0.2 to 0.34 weak correlation [35].

The ‘known-group’ method was used to examine the discriminative validity of the EQ-5D and SF-6D based on its ability to discriminate among patients with different subgroups [13,36]. Patients were grouped according to socioeconomic status, duration of CAD, presence of other medical conditions and the EQ-VAS. We classified the EQ-VAS scores into four groups, namely<65 (bad), 65 to 79 (fair), 80 to 89 (good), and 90 to 100 (excellent) [37]. Subjects with poorer health status were hypothesized to have lower utility scores for these two instruments. Nonparametric Mann–Whitney U tests were performed to identify statistically significant effects of dichotomous variables on utility scores, while Kruskal-Wallis H tests for polychromous variables.

#### Discriminative capacity of the EQ-5D and SF-6D

Ceiling and floor effects (proportion of respondents with the best and worst possible theoretical scores, respectively) were calculated for the EQ-5D and SF-6D. Ceiling and floor effects were considered small if ≤15% of patients occupy the best or worst health states, respectively, and serious if >15% of patients occupy these states [18].

The discriminative capacity of the EQ-5D and SF-6D instruments to detect clinically relevant differences among stable angina patients were compared using the effect size (ES), relative efficiency (RE) statistics, and receiver operating characteristic (ROC) curves. The ROC curve procedure provides a useful method of evaluating the performance of measures against external indicators of health status. The utility measure that generates the largest area under the ROC curve is regarded as the most sensitive at detecting differences in the external indicator. A measure with perfect discrimination would generate an area under the curve (AUC) score of 1.0, whilst a measure with less discriminatory power would generate an AUC score of less than 0.5 [30]. In this analysis, the performance of the EQ-5D and SF-6D was evaluated against the five dimensional scales of the SAQ as an external indicator of health status. Scores for each scale were divided into two groups (>= 50 and<50) indicating better cardiac functioning and worse functioning [38]. ES was used to define the discriminative capacity, and was computed as the difference between the mean of the two groups mentioned above, divided by the pooled standard deviation. The pooled standard deviation was estimated from the corrected standard errors and the weighted number of individuals in the groups [39]. General guidelines define an effect size of 0.2 as small, 0.5 as moderate, and 0.8 as large [40]. This classification was used to interpret differences in the discriminative capacity of the instruments studied. RE statistic is defined as the ratio of the square of the t-statistic of the comparator instrument (assumed to be the SF-6D utility score) over the square of the t-statistic of the reference instrument (assumed to be the EQ-5D utility score). The coefficient higher than 1.0 indicates that the SF-6D is more sensitive than the EQ-5D at detecting differences in external indicators of health with the given sample size, whilst the coefficient lower than 1.0 indicates less sensitivity to detect differences [41].

#### Level of agreement between the EQ-5D and SF-6D

The degree of agreement between utility scores of the EQ-5D and SF-6D was assessed by the intra-class correlation coefficient (ICC) and the Bland-Altman plot. The ICC was computed with the random-effects linear regression model. Coefficients above 0.7 suggest a strong agreement [42]. The paired comparison between the EQ-5D and SF-6D utility scores was made with Wilcoxon’s signed rank test. In the Bland-Altman plot, the average of the two measurements was plotted on the x-axis, and the difference between the two measurements on the y-axis, where the SF-6D was the subtrahend. The deviation of the difference from 0, which implies total agreement, indicates the degree of agreement for each subject on the plot [43].

#### Factors affecting utility difference between the EQ-5D and SF-6D

The factors involved in the variation of the utility difference between the EQ-5D and SF-6D were explored with multiple liner regression (MLR). The utility difference between the EQ-5D and SF-6D was entered as the dependent variable and individual characteristics including age, gender, education, working status, income, BMI, comorbid conditions, disease duration, SAQ scores, and the EQ-VAS for global health status were treated as independent variables.

All data were entered into a database using EpiData (Epidata version 3.1, Epidata Association, Odense, Denmark) and analyzed using STATA 10.0 (STATA Corp LP, Texas, USA).

## Results

### Characteristics of patients

We obtained 411 valid answers from 423 participants with a response rate of 97.16% (Table 1). Half of the patients were women (50.36%), the mean age was 68.08 (11.35) years, and almost 25% had less than six years of schooling. 77.86% of the patients were retired. A high percentage of respondents reported comorbidities including hypertension (56.69%), diabetes (25.30%), and hyperlipidemia (21.17%). Except for angina stability, the mean scores of other SAQ subscales were higher than 50, indicating better functioning. The mean (SD) scores were 0.78 (0.15) for the EQ-5D, 0.68 (0.12) for the SF-6D and 71.23 (12.35) for the EQ-VAS.

**Table 1:** **Sociodemographics and characteristics of Chinese patients with stable angina (N= 411)**

**Sociodemographic**	**N (%)**	**Clinical**	**N (%)**
**Age** (Mean ± SD)	68.08 ± 11.35	**BMI** (Mean ± SD)	24.10 ± 3.76
**Female**	207 (50.36)	**Presence of acute medical condition**	64 (15.57)
**Education**		**With hypertension**	233 (56.69)
Bachelor and above	41 (9.98)	**With diabetes mellitus**	104 (25.30)
High school	144 (35.04)	**With hyperlipidemia**	87 (21.17)
Middle school	125 (30.41)	**Years with CAD** (Mean ± SD)	6.86 **±** 7.13
Primary and below	101 (24.57)	**Patient sources**	
**Working status**		Inpatients	140 (34.06)
Working	50 (12.17)	Outpatients	92 (22.38)
Retired	320 (77.86)	Home	179 (43.55)
Others	41 (9.98)	**SAQ** (Mean ± SD)	
**Marriage status**		Physical limitation	61.80 ± 13.58
Single	3 (0.73)	Angina stability	40.45 ± 34.64
Married	320 (77.86)	Angina frequency	66.40 ± 26.84
Divorced/ Widowed	88 (21.41)	Treatment satisfaction	61.56 ± 14.42
**Monthly household income**		Disease perception	58.33 ± 15.89
<=2500	102 (24.82)	**EQ-VAS score**	71.23 ± 12.35
(2500–4500)	183 (44.53)	**EQ-5D utility score**	0.78 ± 0.15
[4500–10000)	109 (26.52)	**SF -6D utility score**	0.68 ± 0.12
>= 10000	17 (4.14)		
**Tianjin**	212 (51.58)		

CAD: Coronary artery disease; SAQ: Seattle Angina Questionnaire; EQ-VAS: EuroQol visual analog scale; EQ-5D: EuroQol-5D; SF-6D: Short form-6D.

### Construct validation

Convergent validity was demonstrated by the moderate to strong correlation coefficients (range: 0.368-0.594, P< 0.001) for five of eight *a priori* hypotheses in both the EQ-5D and the SF-6D (Table 2). Correlations between the utility scores from these two instruments with the scores for SAQ angina stability were weak, while the correlations between utility and the SAQ physical limitation, SAQ disease perception, and the EQ-VAS scores were relatively strong. Meanwhile, the SAQ physical limitation score correlated strongly with the EQ-5D usual activities and the SF-6D physical function.

**Table 2:** **Correlations between EQ-5D or SF-6D and SAQ or EQ-VAS**

	**SAQ**					**EQ-VAS** ^**a**^
	**Physical limitation** ^**a**^	**Angina stability** ^**a**^	**Angina frequency** ^**a**^	**Treatment satisfaction** ^**a**^	**Disease perception** ^**a**^	
**EQ-5D**						
Utility ^a^	0.496***	0.243***	0.313***	0.281***	0.410***	0.455***
Mobility	−0.303***	0.080	0.068	0.036	−0.030	−0.030
Self-care	−0.322***	0.123*	0.119*	0.106*	0.002	−0.001
Usual activities	−0.594***	−0.320***	−0.229***	−0.392***	−0.496***	−0.326***
Pain/discomfort	−0.131**	−0.318***	−0.391***	-0.271***	−0.347***	−0.388***
Anxiety/depressed	−0.283***	−0.238***	−0.356***	−0.287***	−0.370***	−0.528***
**SF-6D**						
Utility ^a^	0.553***	0.313***	0.365***	0.404***	0.511***	0.470***
Physical function	−0.491***	0.036	−0.147**	−0.016	−0.132**	−0.248***
Role limitation	−0.446***	−0.168***	−0.315***	−0.331***	−0.315***	−0.324***
Social function	−0.531***	−0.223***	−0.235***	−0.269***	−0.364***	−0.313***
Pain	−0.523***	−0.366***	−0.368***	−0.386***	−0.560***	−0.381***
Mental health	−0.227***	−0.297***	−0.370***	−0.366***	−0.395***	−0.494***
Vitality	−0.338***	−0.355***	−0.372***	−0.353***	−0.414***	−0.543***

*P< 0.05 (two-tailed); **P< 0.01 (two-tailed); ***P< 0.001 (two-tailed).

^a^For these variables, higher scores indicate better health, while for other variables higher scores indicate worse health.

Hypothesized moderate-to-strong correlations were bolded.

Table 3 presents the univariate analyses for the SF-6D and EQ-5D utility scores within subgroups. Hypothesis for known-group discriminative validity was confirmed by the differences in utility scores among groups with different health status measured by EQ-VAS. Moreover, both measures discriminated between female and male. Another significant difference in the SF-6D was observed among patients with different education levels, whereas in the EQ-5D, significant difference was observed for the presence of acute medical conditions.

**Table 3:** **Univariate analyses for SF-6D and EQ-5D utility scores within subgroups**

	**N (%)**	**EQ-5D**		**SF-6D**	
		**Mean (SD)**	***P***	**Mean (SD)**	***P***
**Age (Years)**			0.062		0.552
<=44	12 (2.92)	0.79 (0.12)		0.65 (0.13)	
(45--60)	92 (22.38)	0.79 (0.14)		0.67 (0.12)	
(61--74)	170 (41.36)	0.80(0.14)		0.68 (0.12)	
>= 75	137 (33.33)	0.76 (0.16)		0.68 (0.11)	
**Gender**			0.001		0.000
Female	207 (50.36)	**0.77 (0.13)**		**0.66 (0.11)**	
Male	204 (49.64)	**0.80 (0.16)**		**0.70 (0.12)**	
**Education**			0.069		0.007
Bachelor and above	41 (9.98)	0.81(0.15)		**0.72 (0.10)**	
High school	144 (35.04)	0.80 (0.13)		**0.69 (0.11)**	
Middle school	125 (30.41)	0.78 (0.16)		**0.67 (0.12)**	
Primary and below	101 (24.57)	0.75 (0.15)		**0.65 (0.12)**	
**Monthly Household income**			0.127		0.074
<=2500	102 (24.82)	0.76 (0.18)		0.65 (0.13)	
(2500–4500)	183 (44.53)	0.79 (0.13)		0.69 (0.11)	
[4500–10000)	109 (26.52)	0.79 (0.15)		0.67 (0.11)	
>= 10000	17 (4.14)	0.86 (0.10)		0.72 (0.09)	
**Years with CAD**			0.489		0.319
<1	65 (15.82)	0.78 (0.10)		0.67 (0.11)	
[1–5)	143 (34.79)	0.80 (0.14)		0.69 (0.12)	
[5–10)	74 (18.00)	0.78 (0.17)		0.67 (0.11)	
>= 10	129 (31.39)	0.77 (0.16)		0.67 (0.11)	
**Presence of acute medical condition**			0.001		0.106
Yes	64 (15.57)	**0.74 (0.11)**		0.66 (0.13)	
No	347 (84.43)	**0.79 (0.15)**		0.68 (0.11)	
**Presence of chronic medical condition**			0.066		0.574
Yes	338 (82.24)	0.78 (0.15)		0.68 (0.12)	
No	73 (17.76)	0.81 (0.11)		0.67 (0.10)	
**EQ-VAS**			0.000		0.001
<65	102 (24.82)	**0.70 (0.15)**		**0.59 (0.11)**	
(65, 79)	162 (39.42)	**0.78 (0.12)**		**0.68 (0.11)**	
(80, 89)	120 (29.20)	**0.83 (0.15)**		**0.72 (0.11)**	
(90, 100)	27 (6.57)	**0.92 (0.10)**		**0.78 (0.08)**	

The data in boldface mean P< 0.05.

CAD: Coronary artery disease; EQ-VAS: EuroQol visual analog scale; EQ-5D: EuroQol-5D; SF-6D: Short form-6D.

### Ceiling/floor effects for the EQ-5D and SF-6D

There was ceiling effect for the EQ-5D utility score (15.57%) and no patient scored at the ceiling of the SF-6D. However, serious ceiling effects existed in all domains of the EQ-5D, and the largest ceiling effect were observed for mobility (84.18%) and self-care (86.62%) domains. High ceiling effects were also observed in the social function domain (29.20%) and role limitation (26.52%) of the SF-6D. No patient scored at the floor of the EQ-5D utility, and 0.24% scored at the floor of the SF-6D utility. Floor effects were negligible on most domains, except for role limitation (21.17%) and vitality (17.03%) from the SF-6D.

The distribution of responses who reported limitations on the SF-6D dimensions was 15.57% among individuals who reported no limitations on all the EQ-5D dimensions. In this group, a majority of individuals were classified as level 1 for the SF-6D dimension of role limitation (68.75%) and social function (67.19%). Nevertheless, 96.87% of the respondents were classified as level 2 or higher in the physical functioning dimension, 87.50% of the respondents in the vitality dimension, 82.81% of the respondents in the mental health dimension, and 65.62% of the respondents in the pain dimension.

### Sensitivity of the EQ-5D and SF-6D

Table 4 presents effect sizes (ES), relative efficiency (RE) statistics, and area scores under the receiver operating characteristic curves (AUC) for the EQ-5D and SF-6D utility scores between groups based on the dichotomous health status variables. Differences between the five groups for the SF-6D utility scores were large, with ES ranging from 0.573 to 1.179. Most effect sizes on the EQ-5D were moderate or large (ranging from 0. 426 to 1.126).

**Table 4:** **Efficiency of the EQ-5D and SF-6D to detect clinically relevant difference**

**Measure**	**SAQ**	**N**	**Mean (SD)**	**Effect size** ^**a**^	**t Test**		**RE** ^**b**^	**ROC curve**	
					**t statistic**	**P**		**AUC**	**95%CI**
**EQ-5D**	**Physical limitation**								
	>= 50	351	0.81 (0.13)	1.126*	8.062	0.000	1.000	0.762*	(0.690, 0.834)
	<50	60	0.65 (0.20)						
**SF-6D**	**Physical limitation**								
	>= 50	351	0.69 (0.11)	0.981*	7.020	0.000	0.758	0.765*	(0.710, 0.820)
	<50	60	0.59 (0.10)						
**EQ-5D**	**Angina stability**								
	>= 50	232	0.82 (0.14)	0.587*	5.901	0.000	1.000	0.660*	(0.607, 0.712)
	<50	179	0.74 (0.14)						
**SF-6D**	**Angina stability**								
	>= 50	232	0.71 (0.11)	0.706*	7.100	0.000	1.448	0.694*	(0.643, 0.744)
	<50	179	0.63 (0.11)						
**EQ-5D**	**Angina frequency**								
	>= 50	313	0.80 (0.16)	0.426*	3.677	0.000	1.000	0.662*	(0.606, 0.717)
	<50	98	0.74 (0.10)						
**SF-6D**	**Angina frequency**								
	>= 50	313	0.69 (0.11)	0.573*	4.953	0.000	1.814	0.661*	(0.601, 0.722)
	<50	98	0.63 (0.11)						
**EQ-5D**	**Treatment satisfaction**								
	>= 50	323	0.80 (0.15)	0.495*	4.120	0.000	1.000	0.664*	(0.609, 0.719)
	<50	88	0.73 (0.12)						
**SF-6D**	**Treatment satisfaction**								
	>= 50	323	0.70 (0.11)	0.778*	6.466	0.000	2.463	0.724*	(0.667, 0.781)
	<50	88	0.61 (0.10)						
**EQ-5D**	**Disease perception**								
	>= 50	318	0.81 (0.14)	0.707*	5.998	0.000	1.000	0.696*	(0.640, 0.751)
	<50	93	0.71 (0.13)						
**SF-6D**	**Disease perception**								
	>= 50	318	0.71 (0.11)	1.179*	9.997	0.000	2.778	0.814*	(0.767, 0.860)
	<50	93	0.58 (0.10)						

*P< 0.001. For ROC curve, P< 0.001 indicates that AUC statistically significantly greater than 0.5.

^a^Effect sizes were computed as the difference between the means of the groups divided by the pooled standard deviation.

^b^Reference is EQ-5D measure.

EQ-5D: EuroQol-5D; SF-6D: Short form-6D; SAQ: Seattle Angina Questionnaire; SD: standard deviation; ROC: receiver operating characteristics; RE: relative efficiency; AUC: area under ROC curves; CI: confidence interval.

Statistically significant differences (P< 0.001) were found for all between-group comparisons on both the EQ-5D and SF-6D utility scores. RE statistic calculation showed that the EQ-5D was found to be 24.2% more efficient at detecting differences between groups with physical limitations. While when subjects were categorized in terms of angina stability and angina frequency, the SF-6D was 44.8% and 81.4% more efficient than the EQ-5D. When subjects were categorized in terms of treatment satisfaction and disease perception, the SF-6D was 146.3% and 177.8% more efficient than the EQ-5D.

The AUC scores generated by the ROC curves provided a further indication of the sensitivity of the two instruments. The AUC scores of both instruments above 0.5 with statistical significance suggested that the instruments were able to detect the difference between patients with better and worse functioning in the five domains of the SAQ. Except for angina frequency, the SF-6D generated higher AUC scores than the EQ-5D, indicating greater discriminatory power.

### Level of agreement between the EQ-5D and SF-6D

**Figure 1:**
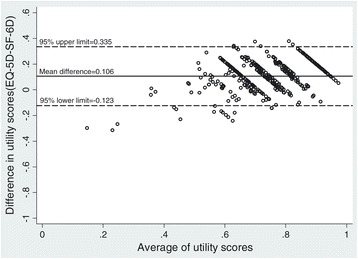
**Bland-Altman plot of difference in utility scores between EQ-5D and SF-6D.**

The degree of agreement between the scores of EQ-5D and SF-6D was assessed by the Bland-Altman plot and by computing an intra-class correlation coefficient (ICC). Poor agreement between the EQ-5D and SF-6D utility scores was observed with a low ICC of 0.448. Wilcoxon’s signed rank test showed that the difference was significant (*P<* 0.001). Bland-Altman analysis indicated lack of agreement between the two measures with the mean difference of 0.106 (Figure 1). The analysis indicated that the 95% limits of agreement between the EQ-5D and SF-6D ranged from −0.123 to 0.335 and over 95% points lay within those limits. A systematic variation was observed, with higher SF-6D at lower mean utility, and lower SF-6D at higher mean utility scores.

### Factors affecting utility difference between the EQ-5D and SF-6D

Table 5 presents the results of the multiple linear regression analyses with the difference between the EQ-5D and SF-6D as the dependent variable. The dependent variable is normally distributed and the multiple linear regression analysis did not obviously break the standard assumptions of linear regression analysis. The values of the VIF (Variance Inflation Factor) are generally below 2 and always below 4, so there is no indication of high multicollinearity [44]. The results found that presence of acute medical conditions significantly influence the difference of the EQ-5D and SF-6D; however, the magnitude of the influence was not large (coefficient 0.043, *P=* 0.008). Similar results were observed for the hypertension (coefficient 0.022, *P=* 0.065). A large magnitude of the influence was observed for inpatient variable (coefficient 0.072, *P=* 0.001). A very small magnitude of the influence was observed for the EQ-VAS variable (coefficient 0.001, *P=* 0.040).

**Table 5:** **Multiple linear regression analyses for utility difference between the EQ-5D and SF-6D**

**Independent variables**	**Utility difference** ^**#**^	
	**Coefficient (95%CI)**	***P***
**Age**	−0.001 (−0.003, 0.001)	0.089
**Male**	−0.010 (−0.033, 0.014)	0.419
**Education (vs. Bachelor and above)**		
High school	0.014 (−0.026, 0.054)	0.491
Middle school	0.034 (−0.007, 0.075)	0.105
Primary and below	0.028 (−0.016, 0.072)	0.211
**Working status (vs. working)**		
Retired	0.006 (−0.033, 0.046)	0.752
Others	0.006 (−0.044, 0.056)	0.807
**Marriage status (vs. married)**		
Single	−0.042 (−0.172, 0.088)	0.525
Divorced/ Widowed	−0.004 (−0.034, 0.026)	0.770
**Monthly Household income (vs. >= 4500)**		
<4500	−0.018 (−0.042, 0.008)	0.173
**BMI**	−0.001 (−0.004, 0.002)	0.453
**Presence of acute medical condition (vs. none)**	0.043 (0.011, 0.075)*	0.008
**Hypertension (vs. none)**	0.022 (−0.001, 0.045)	0.065
**Diabetes mellitus (vs. none)**	−0.002 (−0.027, 0.024)	0.897
**Hyperlipidemia (vs. none)**	−0.011 (−0.038, 0.017)	0.439
**Years with CAD**	0.001 (−0.001, 0.002)	0.471
**Patient sources (vs. Home)**		
Inpatients	0.072 (0.032, 0.113)***	0.001
Outpatients	0.042 (0.009, 0.075)**	0.014
**SAQ**		
Physical limitation	0.0007 (−0.0002, 0.002)	0.158
Angina stability	0.0003 (−0.0001, 0.0008)	0.129
Angina frequency	0.00001 (−0.0005, 0.001)	0.964
Treatment satisfaction	−0.0003 (−0.001, 0.001)	0.516
Disease perception	0.0003 (−0.001, 0.001)	0.518
**EQ-VAS**	0.001 (0.00005, 0.002)*	0.040
**R** ^**2**^	0.119	0.001

^#^SF-6D is the subtrahend; CI: confidence interval.

*P< 0.05; **P< 0.01; ***P< 0.001.

## Discussion

The evidence of validity and sensitivity of the EQ-5D and SF-6D in Chinese patients with stable angina was provided in this study, which demonstrates that the EQ-5D and SF-6D are valid and sensitive preference-based HRQoL instruments in this patient group. However, the performance of the two instruments was not identical. Our results provide useful information for the choice of preference-based HRQoL instruments for stable angina patients. To our knowledge, this is the first comparison study for the EQ-5D and SF-6D among stable angina patients.

In this study, patients from Tianjin and Chengdu were selected as our study sample. Previous evidence has suggested that patient location does not affect the validity of the results [13]. Therefore, samples from the two cities were merged to increase the statistical power and representativeness of study results. Convergent validity was demonstrated by the moderate to strong correlation coefficients with SAQ, a validated instrument for angina, in our study. The correlations between the utilities of the two instruments and two domains of SAQ, physical limitation and disease perception, were relatively strong. This is consistent with the finding that illness perception is correlated with poorer quality of life for cardiac patients [45]. As for ‘known group’ discriminative validity, both the EQ-5D and SF-6D utility scores decreased with poorer health status indicated by the EQ-VAS. Moreover, both measures showed that female patients have lower utility scores than male patients, as previously noted [46]. Furthermore, the results also indicate that increased utility scores are associated with higher education level, but statistical significance is only achieved in the SF-6D. This is consistent with previous studies indicating that lower socioeconomic status is correlated with poorer outcomes in patients with chronic diseases, including cardiac patients [47,48].

Consistent with previous studies, ceiling effects existed in the EQ-5D [13,20,49]. A total of 64 individuals (15.57%) reported no limitations on all the EQ-5D dimensions, while no patients were classified in full health on the SF-6D. Based on the SF-6D responses, individuals reporting full health on the EQ-5D may still have problems on physical function, vitality and mental health dimensions. This disparity can be attributed to the descriptive system of the SF-6D, in which more response levels for each domain are provided and patients might be more likely to find the best description for their status. In fact, a five-level version of the EQ-5D is under development [50]. Preliminary studies indicated that prototype five-level versions could improve the properties of the three-level in terms of reduced ceiling effects, increased reliability, and improved ability to discriminate between different levels of health [51]. In addition, most of the patients had better cardiac functioning indicated by high SAQ scores, which can also partially explain the strong ceiling effect. Effect sizes (ES), relative efficiency (RE) statistics and AUC scores were used to test the discriminative capacity of the EQ-5D and SF-6D. Both instruments were able to detect the differences between patients with different disease severity as measured by the SAQ. It is shown that the SF-6D had greater discriminatory power to detect clinically relevant difference of stable angina patients. This may be partially explained by the serious ceiling effect of the EQ-5D. Previous studies showed that the EQ-5D would be more suitable for measuring the health of more morbidity while the SF-6D may have a limitation in severe patients [18,49].

The mean EQ-5D score exceeded the mean SF-6D score by 0.106 with significant difference, exceeding minimally important differences (MIDs) of both measures [18]. The magnitude of difference is higher than the differences reported in other disease groups or general population [18,20]. Interestingly, previous comparative studies have estimated that the mean EQ-5D score was higher than the SF-6D when the mean EQ-5D score exceeded 0.740, which was consistent with our results [15,52,53]. Conversely, mean EQ-5D utility was less than mean SF-6D utility when the mean EQ-5D score was less than 0.740 [19,54]. The ICC analyses and Bland-Altman plot revealed the inconsistency of these instruments. There were other differences between the two instruments which may explain the different performance. The recall period of both instruments is different is that ‘today’ for the EQ-5D/VAS versus ‘the last four weeks’ for the SF-6D. Another difference is the descriptive systems and the valuations attached to the health states. The SF-6D includes broader aspects of HRQoL and has more response level for each domain. In the EQ-5D, health status is valued using the time trade-off (TTO) method, whereas the SF-6D assigns value to health states using the standard gamble (SG) [55]. Also, in the specific China scoring algorithm of the EQ-5D, if any dimension is at level 3, a N3 term will be included. The existence of N3 term in the China scoring algorithm could be one of the reasons for the discrepancy between EQ-5D and SF-6D. According to our results, the SF-6D is shown to be more appropriate choice among stable angina patients because of its higher sensitivity and lower ceiling effect.

Our study had several limitations. The first was that as a cross-section study, we did not examine the longitudinal response and reliability of the EQ-5D and SF-6D, for which are important psychometric characteristics of instruments. Secondly, the relatively small sample size of severe stable angina patients might aggregate the ceiling effect. Further studies with a larger sample size are warranted. Third, similar to some previous studies, there were no objective groups in our known-group analysis. All comparisons are relative because there was no ‘gold standard’ objective measure to compare the measures with.

## Conclusions

The EQ-5D and SF-6D are demonstrated to be valid and sensitive preference-based HRQoL instruments in Chinese stable angina patients. The SF-6D may be a superior to the EQ-5D, with a lower ceiling effect and greater sensitivity. Further study is needed to compare other properties, such as reliability and longitudinal response.

## References

[1] Earnshaw J, Lewis G (2008). NICE guide to the methods of technology appraisal: pharmaceutical industry perspective. Pharmacoeconomics.

[2] Sullivan SD, Lyles A, Luce B, Grigar J (2001). AMCP guidance for submission of clinical and economic evaluation data to support formulary listing in U.S. health plans and pharmacy benefits. J Manag Care Pharm.

[3] Drummond MF, Sculpher MJ, Torrance GW, O’Brien BJ, Stoddart GL (2005). Methods for the Economic Evaluation of Health Care Programmes.

[4] Kopec JA, Willison KD (2003). A comparative review of four preference-weighted measures of health-related quality of life. J Clin Epidemiol.

[5] Neumann PJ, Goldie SJ, Weinstein MC (2000). Preference-based measures in economic evaluation in health care. Annu Rev Public Health.

[6] Kaplan RM, Bush JW, Berry CC (1976). Health status: types of validity and the index of wellbeing. Health Serv Res.

[7] Torrance GW, Furlong W, Feeny D, Boyle M (1995). Multi-attribute preference functions. Health utilities index. Pharmacoeconomics.

[8] Rabin R, De Charro F (2001). EQ-5D: a measure of health status from the EuroQol Group. Ann Med.

[9] Hawthorne G, Richardson J, Day NA (2001). A comparison of the assessment of quality of life (AQoL) with four other generic utility instruments. Ann Med.

[10] Brazier J, Roberts J, Deverill M (2002). The estimation of a preference-based measure of health from the SF-36. J Health Econ.

[11] Bansback N, Tsuchiya A, Brazier J, Anis A (2012). Canadian valuation of EQ-5D health states: preliminary value set and considerations for future valuation studies. PLoS One.

[12] Liu GG, Wu H, Li M, Gao C, Luo N (2014). Chinese time trade-off values for EQ-5D health states. Value Health.

[13] Zhao FL, Yue M, Yang H, Wang T, Wu JH, Li SC (2010). Validation and comparison of EuroQol and short form 6D in chronic prostatitis patients. Value Health.

[14] Kontodimopoulos N, Argiriou M, Theakos N, Niakas D (2011). The impact of disease severity on EQ-5D and SF-6D utility discrepancies in chronic heart failure. Eur J Health Econ.

[15] Van Stel HF, Buskens E (2006). Comparison of the SF-6D and the EQ-5D in patients with coronary heart disease. Health Qual Life Outcomes.

[16] Obradovic M, Lal A, Liedgens H (2013). Validity and responsiveness of EuroQol-5 dimension (EQ-5D) versus Short Form-6 dimension (SF-6D) questionnaire in chronic pain. Health Qual Life Outcomes.

[17] Mulhern B, Meadows K (2014). The construct validity and responsiveness of the EQ-5D, SF-6D and Diabetes Health Profile-18 in type 2 diabetes. Health Qual Life Outcomes.

[18] Harrison MJ, Davies LM, Bansback NJ, McCoy MJ, Verstappen SM, Watson K, Symmons DP, British Society for Rheumatology Biologics Register Control Centre Consortium (2009). The comparative responsiveness of the EQ-5D and SF-6D to change in patients with inflammatory arthritis. Qual Life Res.

[19] Lamers LM, Bouwmans CA, van Straten A, Donker MC, Hakkaart L (2006). Comparison of EQ-5D and SF-6D utilities in mental health patients. Health Econ.

[20] Cunillera O, Tresserras R, Rajmil L, Vilagut G, Brugulat P, Herdman M, Mompart A, Medina A, Pardo Y, Alonso J, Brazier J, Ferrer M (2010). Discriminative capacity of the EQ-5D, SF-6D, and SF-12 as measures of health status in population health survey. Qual Life Res.

[21] The Task Force on the Management of stable angina pectoris of the European Society of Cardiology, European Society of Cardiology (ESC) (2006). Guidelines on the Management of Stable Angina Pectoris.

[22] Center for Health Statistics and Information, Ministry of Health (2008). An Analysis Report of National Health Services Survey in China.

[23] Kannel WB, Feinleib M (1972). Natural history of angina pectoris in the Framingham study: Prognosis and survival. Am J Cardiol.

[24] Gandjour A, Lauterbach KW (1999). Review of quality-of-life evaluations in patients with angina pectoris. Pharmacoeconomics.

[25] Spertus JA, Winder JA, Dewhurst TA, Deyo RA, Prodzinski J, McDonell M, Fihn SD (1995). Development and evaluation of the Seattle Angina Questionnaire: a new functional status measure for coronary artery disease. J Am Coll Cardiol.

[26] Wilson A, Wiklund I, Lahti T, Wahl M (1991). A summary index for the assessment of quality of life in angina pectoris. J Clin Epidemiol.

[27] Ware JE, Snow KK, Kosinski M, Gandek B (1993). SF-36 Health Survey Manual and Interpretation Guide.

[28] Hunt SM, McKenna SP, McEwen J, Backett EM, Williams J, Papp E (1980). A quantitative approach to perceived health status: a validation study. J Epidemiol Community Health.

[29] McGillion MH, Croxford R, Watt-Watson J, Lefort S, Stevens B, Coyte P (2008). Cost of illness for chronic stable angina patients enrolled in a self-management education trial. Can J Cardiol.

[30] Streiner DL, Norman GR (2008). Health Measurement Scales: A Practical Guide to Their Development and Use.

[31] Wang H, Kindig DA, Mullahy J (2005). Variation in Chinese population health related quality of life: results from a EuroQol study in Beijing, China. Qual Life Res.

[32] Shi JF, Kang DJ, Qi SZ, Wu HY, Liu YC, Sun LJ, Li L, Yang Y, Li Q, Feng XX, Zhang LQ, Li J, Li XL, Yang Y, Niyazi M, Xu AD, Liu JH, Xiao Q, Li LK, Wang XZ, Qiao YL (2012). Impact of genital warts on health related quality of life in men and women in mainland China: a multicenter hospital-based cross-sectional study. BMC Public Health.

[33] Lam CL, Brazier J, McGhee SM (2008). Valuation of the SF-6D health states is feasible, acceptable, reliable, and valid in a Chinese population. Value Health.

[34] Liu XT, Kong SP, Liao ZY, Sike L (1997). Asessment study on physical function and the quality of life for CHD patients with SAQ. Chin Behav Sci.

[35] Spilker B (1996). Quality of Life and Pharmacoeconomics in Clinical Trials.

[36] Jin H, Wang B, Gao Q, Chao J, Wang S, Tian L, Liu P (2012). Comparison between EQ-5D and SF-6D utility in rural residents of Jiangsu Province, china. PLoS One.

[37] Barton GR, Sach TH, Avery AJ, Jenkinson C, Doherty M, Whynes DK, Muir KR (2008). A comparison of the performance of the EQ-5D and SF-6D for individuals aged > or= 45 years. Health Econ.

[38] Berra K, Fletcher B, Miller NH (2008). Chronic stable angina: Addressing the needs of patients through risk reduction, education and support. Clin Invest Med.

[39] Martins WP, Zanardi JV (2011). Subgroup analysis and statistical power. Eur J Obstet Gynecol Reprod Biol.

[40] Kazis LE, Anderson JJ, Meenan RF (1989). Effect sizes for interpreting changes in health status. Med Care.

[41] Fayers P, Machin D (2000). Quality of Life: Assessment, Analysis, and Interpretation.

[42] Rabe-Hesketh S, Everitt (2006). A Handbook of Statistical Analyses Using Stata.

[43] Bland JM, Altman DG (1986). Statistical methods for assessing agreement between two methods of clinical measurement. Lancet.

[44] Mason CH, Perreault WD (1991). Collinearity, power, and interpretation of multiple regression analysis. J Mark Res.

[45] Le Grande MR, Elliott PC, Worcester MU, Murphy BM, Goble AJ, Kugathasan V, Sinha K (2012). Identifying illness perception schemata and their association with depression and quality of life in cardiac patients. Psychol Health Med.

[46] Kimble LP, McGuire DB, Dunbar SB, Fazio S, De A, Weintraub WS, Strickland OS (2003). Gender differences in pain characteristics of chronic stable angina and perceived physical limitation in patients with coronary artery disease. Pain.

[47] Sykes DH, Hanley M, Boyle DM, Higginson JD, Wilson C (1999). Socioeconomic status, social environment, depression and postdischarge adjustment of the cardiac patient. J Psychosom Res.

[48] Kington RS, Smith JP (1997). Socioeconomic status and racial and ethnic differences in functional status associated with chronic diseases. Am J Public Health.

[49] Bharmal M, Thomas J (2006). Comparing the EQ-5D and the SF-6D descriptive systems to assess their ceiling effects in the US general population. Value Health.

[50] Herdman M, Gudex C, Lloyd A, Janssen M, Kind P, Parkin D, Bonsel G, Badia X (2011). Development and preliminary testing of the new five-level version of EQ-5D (EQ-5D-5 L). Qual Life Res.

[51] Janssen MF, Pickard AS, Golicki D, Gudex C, Niewada M, Scalone L, Swinburn P, Busschbach J (2013). Measurement properties of the EQ-5D-5 L compared to the EQ-5D-3 L across eight patient groups: a multi-country study. Qual Life Res.

[52] Brazier J, Roberts J, Tsuchiya A, Busschbach J (2004). A comparison of the EQ-5D and SF-6D across seven patient groups. Health Econ.

[53] Petrou S, Hockley C (2005). An investigation into the empirical validity of the EQ-5D and SF-6D based on hypothetical preferences in a general population. Health Econ.

[54] Longworth L, Bryan S (2003). An empirical comparison of EQ-5D and SF-6D in liver transplant patients. Health Econ.

[55] Green C, Brazier J, Deverill M (2000). Valuing health-related quality of life. A review of health state valuation techniques. Pharmacoeconomics.

